# Prevalence of Overweight, Obesity, Abdominal Obesity, and Obesity-Related Risk Factors in Polish Preschool Children: A Cross-Sectional Study

**DOI:** 10.3390/jcm10040790

**Published:** 2021-02-16

**Authors:** Piotr Matłosz, Justyna Wyszyńska, Muhammad Asif, Agnieszka Szybisty, Muhammad Aslam, Artur Mazur, Jarosław Herbert

**Affiliations:** 1 Institute of Physical Culture Sciences, Medical College, University of Rzeszów, ul. Cicha 2a, 35-326 Rzeszów, Poland; agnieszkaszybisty@wp.pl (A.S.); jherbert@ur.edu.pl (J.H.); 2 Institute of Health Sciences, Medical College, University of Rzeszów, 35-959 Rzeszów, Poland; justyna.wyszynska@onet.pl; 3 Department of Statistics, Govt. Degree College, Qadir Pur Raan, 60000 Multan, Pakistan; asifmalik722@gmail.com; 4 Department of Statistics, Bahauddin Zakariya University, 60000 Multan, Pakistan; aslamasadi@bzu.edu.pk; 5 Institute of Medical Sciences, Medical College, University of Rzeszów, 35-959 Rzeszów, Poland; drmazur@poczta.onet.pl

**Keywords:** preschool children, obesity, body fat percentage, abdominal obesity, risk factors

## Abstract

The aim of this study was to assess the prevalence of overweight, obesity, abdominal obesity (AO), and obesity-related risk factors in children aged 5–6 years from Poland. The study was conducted at 22 randomly selected kindergartens representing each city district. A cross-sectional study of 1172 children aged 5–6 years was conducted using questionnaire forms and physical measurements. The physical measurements included body height, weight, waist circumference (WC), and body fat percentage (BFP). A univariate and multivariate logistic regressions were performed to evaluate the risk factors for excess weight, excess adiposity, and abdominal obesity (AO). The prevalence of excess weight (BMI ≥ 85th percentile) was 11.0%. The prevalence of excess adiposity (BFP ≥ 85th percentile) was 42.3%. Prevalence of AO (WC ≥ 90th percentile) was higher in girls compared to boys (14.9% vs. 10.7%, respectively). Multivariate logistic regression analysis indicated that children whose both parents were obese had significantly higher risk of excess weight, excess adiposity, and AO. Lower education level of fathers was associated with higher risk of excess weight and excess adiposity in children, while a lower level of maternal education was associated with higher risk of AO in children. Screen time over 120 min per day, participating less than once a week in at least 60 min of moderate-to-vigorous physical activity (MVPA) and birth weight over 4000 g were associated with excess weight, excess adiposity and AO. Moreover, cesarean delivery was associated with higher risk of excess weight and excess adiposity, and lower socio-economic status with higher risk of AO. This study revealed that excess adiposity and AO differed by gender. Parental obesity, screen time, MVPA, and birth weight could be significant determinants of excess weight, excess adiposity and AO in Polish preschool children.

## 1. Introduction

Increase in the prevalence of childhood obesity and overweight is a general public health concern. Worldwide, over 38 million children under five years of age are estimated to have overweight or obesity [1]. It has been shown that early childhood obesity track into adulthood [2]. This have important public health consequences since excess body mass is associated among others with increased risk for non-communicable diseases like type 2 diabetes, hypertension, cardiovascular diseases, and specific types of cancer. Furthermore, childhood obesity is an independent predictor of overall mortality; thus, could affect life expectancy of the youngest population [3]. Therefore, prevention initiatives implementing in early childhood to reduce associated short- and long-term morbidity, should be a major public health priority. Evensen et al. concluded that preventive and treatment strategies among children at high risk of over fatness should start before 5–7 years of age [4].

Abdominal obesity (AO), also known as central obesity, is understood as the excessive accumulation of fat tissue in the abdominal region. The most widely accepted method and the simplest clinical measure of AO, since it is non-invasive, quick and easy to obtain remains waist circumference (WC) measurement. For pediatric population, due to the continuous growth and development process, WC measurements should be categorized according to age and gender-specific centile chart. In children and adolescents, AO is defined as WC for age and gender greater than or equal to the 90th percentile [5]. In children, AO is a better predictor of cardiovascular risk factors than general obesity measured by the body mass index (BMI) [6]. The Bogalusa Heart Study indicated that the distribution of central fat determined by WC in children aged from 5 to 17 years is associated with abnormal concentrations of triglycerides, low density lipoprotein, high density lipoprotein, and insulin [7]. Moreover, it has been recognized that individuals with a similar BMI can vary considerably in their abdominal fat mass. A substantial proportion of children with BMI indicating normal body mass, may have a disproportionately large WC and may suffer from AO [8,9]. Furthermore, appears that the occurrence of AO has increased more rapidly in recent decades than the occurrence of obesity defined according to BMI [10,11]. However, neither BMI nor WC cannot distinguish between fat and fat-free mass. Moreover, elevated BMI not always reflect increased adiposity. Individuals with excessive muscle growth can show a high BMI without having an excess of fat, and may be misjudged to be obese [9]. One of the reliable and accurate methods for the measurement of fat tissue content is bioelectrical impedance analysis (BIA). In addition, this method is quick, non-invasive and relatively inexpensive [12]. It was indicated that BIA device owed strong significant correlations with dual-energy X-ray absorptiometry fat mass, body fat percentage (BFP), and total fat-free mass (FFM) [13].

Several factors have been discussed that may contribute to obesity prevalence. Existing evidence on the associations between socio-demographic variables with overweight and obesity in school-aged children is inconsistent. Moreover, findings seem to be influenced by the obesity definition applied. Wang indicated that in developing countries socioeconomic status is positively related to childhood obesity, whereas in developed countries children with higher socioeconomic status are less likely to be obese [14]. Association between socioeconomic factors and body mass status is heterogeneous across different European regions. Results from the multi-Centre IDEFICS study, included 11,994 2-to-9-year-old children, showed an inverse relationship between socioeconomic status and prevalence of overweight and obesity in 5 out of the 8 participating European countries [15]. However, findings from the national longitudinal study of Irish children, showed no difference in obesity prevalence across social classes in children aged three years old [16].

Further, literature suggests that obesity usually results from a complex interaction of genetic and lifestyle factors [17]. Few studies have tried to assess complete picture of risk factors of excess body mass in children; however, most studies have evaluated fewer predictors of obesity, such as parental obesity [18], perinatal factors [19,20], physical activity [21], screen time [22], diet [23], and sleep duration [24]. However, it is accepted that usually there is no single cause of obesity, but development of childhood obesity is multifactorial [25]. Assessment of obesity risk factors still remains under-investigated among Polish preschool children.

Poland is recognized by the United Nations as a very highly developed country. Poland’s Human Development Index value for 2019 was 0.880, positioning it at 35 out of 189 countries and territories. Gross domestic product per capita is still below the European Union average, but is classified as a high-income economy by the World Bank and ranks 22nd worldwide in terms of nominal gross domestic product [26].

In Poland in 2016, the prevalence of overweight and obesity in boys and girls was 17.2% and 19.1%, respectively, as defined by the Centers for Disease Control and Prevention (CDC) [27]. Globally, one in five children and adolescents is overweight [1]. The study of health behavior of school-age children showed that among 11-year-olds the highest incidence of overweight was recorded in Greece (33%), Portugal (32%), Ireland (30%), and Spain (30%), while the lowest was in the Netherlands (13%) and Switzerland (11%). Prevalence of overweight among 15-year-olds ranged from 10% (in Armenia, Lithuania, and Russia) to 23% (in Greece). In total, 27% of 13-year-olds in Europe were diagnosed overweight [28]. In the younger population, under the age of 10, over 40% of children living in southern Europe was diagnosed with overweight or obese, while in northern Europe less than 10%. Excess body weight was found more often in girls than in boys (21.1% vs. 18.6%, respectively) [29]. Therefore, regarding to the prevalence of the overweight and obesity of preschool children Poland could be considered in the middle rates among other European countries.

Rzeszów is the capital of the Podkarpackie province. As the largest and most dynamically developing city in region with relatively low level of unemployment could be recognized as a city with high socioeconomic potential. Recent study among preschool children living in this city showed that the overweight and obesity was found in 11% and 10.5% of 3–6 years old children, respectively [30].

To the best of our knowledge, no study have assessed the prevalence of obesity, using different obesity definitions, among large sample of children aged 5 to 6 years with identification of socio-demographic, socio-economic, perinatal, and related to lifestyle risk factors. The recognition of risk factors is crucial to develop effective intervention programs for the prevention of childhood overweight and obesity. Therefore, the aim of the present study was to evaluate the prevalence of AO and obesity defined according to BMI and BFP in preschool children aged 5–6 years and to identify socio-demographic, socio-economic, perinatal and related to lifestyle correlates of general and AO.

## 2. Materials and Methods

### 2.1. Study Design and Study Sample

Written informed consent was obtained from parents or legal guardians prior to participation in the study. The study was approved by the Bioethics Committee at the Medical Department of the University of Rzeszów, Poland, and it was conducted in accordance with ethical standards laid down in an appropriate version of the Declaration of Helsinki. The study was conducted from October to November at each year from 2018 to 2019.

In 2018, there were a total of 105 kindergartens in Rzeszów. Aiming to recruit at least 20 institutions, 25 kindergartens was invited to participate by random selection (with the use of random sampling and allocation tool in SPSS 25 software) of one municipal institution per each large city district to ensure representativeness in terms of socioeconomic status and living conditions (city center vs suburbs). The management of 22 institutions agreed to participate in the project. Based on information provided by kindergarten principals regarding the number of 5–6 year old children attending to each institution the total of 1358 invitations to participate were handed over to parents/ legal guardians. The consent of 1224 parents was obtained for child participation in the measurements for the purpose of the present study (response rate—90.1%). Of those respondents, 52 were excluded from the study for the following reasons: a functional state that does not allow for self-maintenance of a standing position (*n* = 1), strong anxiety of examination (*n* = 3), taking medication affecting body composition (*n* = 3), a failure to return or complete all the survey questions (*n* = 45). Ultimately, the study group consisted of 1172 children (48.8% girls).

### 2.2. Anthropometric Measurements

Children’s body mass and height were measured, using standard protocol and equipment which was calibrated before and during the period of data collection. The measurements of children’s body mass using body composition analyzer (BC-420 MA, Tanita, Tokyo, Japan) and height using a portable stadiometer (HR-200, Tanita, Tokyo, Japan) were taken. Body mass index was calculated according to the Quetelet’s equation as body mass /height2 (kg/m^2^). Based on BMI values, the BMI percentile of each participants was calculated using Polish age- and sex-specific BMI charts [31]. According to the Centers for Disease Control and Prevention criteria body mass categories (underweight, normal, overweight, and obesity) based BMI percentile were defined [32]. For the purpose of this study, children were classified into two groups: (1) "normal weight" (BMI percentile < 85th percentile) and (2) "excess weight" (overweight/obesity) (≥85th percentile) [32].

### 2.3. Waist Circumference

Waist circumference was measured directly on the skin with a flexible steel anthropometric tape (Lufkin W606PM, Cooper Industries, Mount Vernon, OH, USA) to the nearest 0.1 cm. Measurements were taken in standing position, at the end of natural expiration at the midpoint between the lowest rib and the iliac crest. Polish gender and age-specific WC percentile charts were used [33]. Normal WC was defined as WC < the 90th percentile, AO was defined as WC ≥ the 90th percentile [5].

### 2.4. Body Fat Percentage

The body composition analyzer (BC-420 MA, Tanita, Tokyo, Japan) was used to estimate the BFP. It was shown that BIA method is a reliable and accurate tool for the measurement of body composition in the pediatric population [12]. Based on the BFP percentiles, underfat (BFP < 2nd percentile), normal (BFP < 85th percentile and ≥ 2nd percentile), overfat (BFP ≥ 85th percentile and < 95th percentile), and obesity (BFP ≥ 95th percentile) were diagnosed [34]. For the purpose of this study, the participants were classified as: (1) "no excess adiposity" (BFP percentile < 85th percentile) and (2) "excess adiposity" (≥ 85th percentile) [34].

### 2.5. Socio-Demographic and Socio-Economic Data

Family socio-economic and socio-demographic characteristics (children’s sex and date of birth, place of residence, and parents’ education) as well as parents body weight and body height were self-reported by the parents/caregivers. Based on the collected information socio-economic status was defined as low, middle, or high. Parental BMI was calculated as underweight (BMI < 18.5), normal (BMI 18.5–24.9), overweight (BMI 25–29.9), and obese (BMI ≥ 30) [35].

### 2.6. Lifestyle Factors

Parents were asked to report children’s screen time and frequency of participation in at least 60 min of moderate-to-vigorous physical activity (MVPA). Screen time was defined as the time spent watching television, smartphone, video games, or computer and was measured as the average number of hours per day spent on screen.

### 2.7. Perinatal Risk Factors

Parents provided information on pregnancy and birth based on child’s health record. The relevant data included the child’s birth weight, duration of pregnancy and mode of delivery (natural childbirth or cesarean section). Birth weight was classified as: low birth weight (<2500 g), normal birth weight (2500–4000 g), and large weight for gestational age (≥4000 g). Duration of pregnancy was classified as: preterm birth <37 weeks, and at term ≥37 weeks [36].

### 2.8. Statistical Analysis

Statistical analysis was performed using the SPSS 25 software (IBM, North Harbour, UK). Statistics are presented as mean (±SD) and *n* (%). The independent t-test was used to determine a statistically significant difference between boys and girls. Associations between the prevalence of body mass categories were determined by Pearson chi-square test. A univariate and multivariate logistic regressions, odds ratio (OR) and adjusted odds ratio (adj. OR) with a 95% confidence interval (CI) were calculated to determine factors associated with excess weight, excess adiposity, and AO. The level of statistical significance was adopted at *p* < 0.05.

## 3. Results

Table 1 shows that the overall prevalence of overweight and obesity defined based on BMI was 6.7% and 4.4%, respectively, and prevalence of AO based on WC was 12.7%. Higher prevalence of unhealthy body mass was observed, while overfat and obesity were defined according to BFP (22.4% and 19.9%, respectively). Based on BMI in boys, 6.5% were overweight and 3.8% were obese. Likewise, in girls, 6.8% was overweight and 4.9% was obese. There was a non-significant tendency regarding the prevalence of overweight and obesity, defined according to BMI, in both gender. The prevalence of overfat and obesity, defined based on BFP was higher both in boys and girls (26.3% and 21.8% in boys, 18.4% and 17.8% in girl, respectively) compared to BMI. Prevalence of AO was higher in girls compared to boys (14.9% vs. 10.7%, respectively).

Table 2 shows the distribution of potential risk factors of childhood obesity according to sex. Differences between boys and girls were found in place of residence, father’s education, frequency of participation in 60 min of MVPA and birth weight. Significantly more boys participated in at least 60 min of MVPA daily compared to girls (29.2% vs. 22.9%). Girls’ birth weight was lower than boys’ (3261.2 g vs. 3400.7 g, respectively).

A univariate and multivariate logistic regression analysis were performed to assess the significant determinants of excess weight (overweight/obesity), defined according to BMI (Table 3). The results showed that fathers’ lower education was associated with excess weight (adj. OR = 1.86, *p* = 0.001). Additionally, children of obese mothers had a greater risk of excess weight (adj. OR = 2.66, *p* = 0.002). The risk of overweight/obesity in children was much higher when both parents were obese (adj. OR = 5.31, *p* < 0.001). Children whose screen time was higher than 120 min daily had two fold higher risk of excess weight compared to peers with screen time less than 60 min per day (adj. OR = 2.00, *p* = 0.045). Moreover, participating less than once a week in at least 60 min of MVPA was also significantly associated with excess weight (adj. OR = 2.65, *p* = 0.003). Birth weight over 4000 g and caesarean birth were associated with significantly higher adj. ORs for excess weight (OR = 3.59, *p* < 0.001 and OR = 1.61, *p* = 0.014, respectively). In all abovementioned potential risk factors of childhood obesity significant results were observed both for adjusted and unadjusted models.

Table 4 shows a univariate and multivariate logistic regression analysis to assess the significant determinants of excess adiposity. The results showed that fathers’ lower education was associated with excess adiposity in children (adj. OR = 1.30, *p* = 0.034). Obesity of mother or father analyzed separately contributed to almost two fold higher risk of excess adiposity in children (adj. OR = 1.95 and 1.85, respectively). More than two times greater risk of excess adiposity among children was observed when both parents were obese (adj. OR = 4.65, *p* < 0.001). Additionally, participants with screen time from 60 to 120 min daily were more likely to be classified with excess adiposity compared with those with screen time lower than 60 min per day (adj. OR = 1.47, *p* = 0.003). Even greater risk of excess adiposity was found in children whose screen time was higher than 120 min per day (adj. OR = 1.93, *p* = 0.014). Moreover, participating less than once a week in at least 60 min of MVPA was significantly associated with excess adiposity (adj. OR = 2.51, *p* = < 0.001). Birth weight over 4000 g and caesarean birth were also associated with significantly higher adjusted ORs for excess weight (adj. OR = 2.08, *p* < 0.001 and adj. OR = 1.29, *p* = 0.037, respectively). Significant results were observed both for adjusted and unadjusted models in all cases except for father’s education and participating in at least 60 min of MVPA for 2–3 times per week where significant results were observed only in adjusted model.

Table 5 shows a univariate and multivariate logistic regression analysis to assess the significant determinants of AO. The results showed that mothers’ lower education was associated with AO in children (adj. OR = 1.97, *p* = < 0.001). Middle or low socio-economic status also contributed to about two fold higher risk of AO (adj. OR = 2.09, *p* = 0.020). Moreover, children of obese fathers’ had a greater risk of AO (adj. OR = 2.05, *p* = 0.014), and the risk of AO in children doubled when both parents were obese (adj. OR = 4.39, *p* < 0.001). Participants with screen time higher than 120 min per day were more likely to be classified with AO compared with those with screen time lower than 60 min per day (adj. OR = 1.99, *p* = 0.040). Additionally, participating less than once a week in at least 60 min of MVPA was significantly associated with excess adiposity (adj. OR = 1.92, *p* = 0.038). Birth weight over 4000 g was associated with significantly higher adjusted OR for AO (adj. OR = 3.56, *p* < 0.001). Significant results were observed both for adjusted and unadjusted models in all cases except for screen time above 120 min per day where significant results were observed only in adjusted model.

A summary of main findings of the study are also presented in the supplementary material in the form of three infographics (Figures S1–S3).

## 4. Discussion

Overall, our findings suggest that children from families of obese parents were significantly more likely to be classified with excess weight, excess adiposity, and AO, than children of parents with normal body weight. Lower parental education level also contributed to excess weight, excess adiposity, and AO in children. Moreover, children with screen time over 120 min per day compared to those with screen time less than 60 min per day, had significantly higher risk of excess weight, excess adiposity, and AO. Similarly, participating in less than once a week in at least 60 min of daily MVPA and birth weight over 4000g contributed to higher risk of excess weight, excess adiposity, and AO. Caesarian delivery was factor associated with excess weight and excess adiposity, while middle or low socio-economic status was factor associated with AO. To the best of our knowledge, this is the first study that evaluated the prevalence of obesity defined by BMI, BFP, and WC and associated factors in a relatively large sample of children aged five to six years in Poland.

Our results showed that the prevalence of obesity in sample of preschool children largely varied depending on the obesity definitions. Overall, the highest prevalence of obesity was observed based BFP measurement (19.9%), lower based WC measurement (12.7%), and the lowest based BMI (4.4%). Discrepancies in the prevalence of obesity defined by BMI and BFP may be explained due to the fact that despite the good reliability of BIA methods in evaluation BFP in children, the validity and measurement error could be unsatisfactory [37]. Moreover, it has been showed that BIA-BFP overestimated DEXA-BFP by a mean of 2.53% (with the limits of agreement of 4.29% and 9.36%) [12]. A previous study on Polish prepubertal children aged 7–9 years showed that the prevalence of obesity, based on International Obesity Task Force standards, were 3.7% among girls and 3.6% among boys in 2001. There was a significant association between the prevalence of obesity in children and their parents (OR from 5.06 in girls to 7.22 in boys). However, neither socio-economic status nor screen time and physical activity level were not significant risk factors for obesity [38]. According to World Health Organization (WHO) 38 million children under the age of 5 were overweight or obese in 2019 [1]. Globally, between 1980 and 2015, the prevalence of obesity increased from 3.9% to 7.2% in boys and from 3.7 to 6.4% in girls aged 2–4 years [39]. A systematic review and meta-regression of studies on prevalence of overweight and obesity among European children aged 2–7 years indicated that the pooled prevalence of excess weight in European children during the period 2006–2016 was 17.9% (95% CI: 15.8–20.0), and the pooled prevalence of obesity was 5.3% (95% CI: 4.5–6.1). Highest prevalence of overweight/obesity was found in Southern European countries [40]. Results regarding obesity prevalence from the ToyBox study conducted among a large Pan-European cohort of preschool children and their families are in line with the results of previous studies [41]. Populations of the ToyBox study with higher prevalence of obesity had the lowest diet quality, the lowest levels of physical activity, and the highest levels of sedentary time [42,43]. These evidence confirm the need for effective prevention and interventions starting as early as infancy, since diet and lifestyle factors are modifiable factors associated with obesity. According to the National Health and Nutrition Examination Survey (NHANES) study, AO was diagnosed in 32.9% of children and adolescents aged 6 to 18 years [44], which implies that it was prevalent 2.5 times more often than in our study (12.7%). Studies demonstrated that the prevalence of AO has increased to a higher degree than general obesity in youth [10,45]. Bogolusa Heart Study indicated that children within normal and overweight classifications with AO had a higher cardiometabolic risk than children with excess weight but without excessive abdominal fat [46].This indicates the need to include WC measurements into routine clinical practice, in addition to measurements of weight and height.

Systematic review and meta-analysis of thirty-seven studies that evaluated 53,521 subjects among countries from almost all continents showed that BMI has high specificity in identifying pediatric obesity, but moderate sensitivity. Pooled results from all analyzed studies showed sensitivity of 73%, suggesting over a quarter of children not labelled as obese by BMI might indeed have excess adiposity [47]. Similar outcomes was found in study among Polish preschool children form Krakow City [48], which combined with present study outcomes could suggest that associations between excess adiposity and excess weight among Polish children stays in line with the findings form other high developed countries.

Results of our study indicated that parental education and weight status were identified as correlates of preschool-aged children’s excess weight, excess adiposity and AO. Moreover the prevalence of excess weight, excess adiposity and AO was found to be higher in children with one or two obese parents (adj. OR from 1.85 to 5.31) and mother of father with middle educational level (adj. OR from 1.30 to 1.97). In addition middle or low socio-economic status compared to high was found to be a risk factor of AO (adj. OR = 2.09). These results are in line with the previous evidence from a large Pan-European cohort of preschool children which also demonstrated that maternal education, maternal weight status, and paternal weight status were identified as factors of preschool-aged children’s excess weight. Children from low socio-economic status mothers had higher risk of excess weight compared with peers from medium- to high- socio-economic status mothers (OR = 1.41). Similarly, excess weight of mothers (OR = 1.64) and fathers (OR = 1.79) were correlates of overweight or obesity in children [41]. Recent systematic review and meta-analysis indicated that the ORs of overweight and obesity were higher in the children with lowest socio-economic status compared to those with highest (OR = 1.10 and OR = 1.41, respectively), and in children with lower parental education level. Increased risk of excess weight in children with lower socio-economic status may be associated with more frequent diets rich in low cost energy dense food, less participation in physical activity or sports, and less interest in healthy weight maintaining [49]. Evidence from multiple countries demonstrated significant relation between parents and children weight status, but this relation varied by child age, type of parent–child pair and weight classification. The meta-analysis suggested strong parent–child obesity association (pooled OR = 2.22). Stronger relationships were found in older children compared to younger, and in both obese parents compared to one [18]. It indicated that families and parents may be a key target for obesity prevention and intervention efforts.

Our study suggested that children who exceeded the recommended screen time of less than 2 h a day were more likely to have excess weight (adj. OR = 2.00), excess adiposity (adj. OR = 1.93) and AO (adj. OR = 1.99) than those who met the recommendation. Moreover, attending in less than once a week in at least 60 min of MVPA was significantly associated with excess weight (adj. OR = 2.65), excess adiposity (adj. OR = 2.51) and AO (adj. OR = 1.99). Positive association between screen time and risk of excess weight is claimed by decreased level of physical activity and increased intake of energy. Recent meta-analysis showed that children and adolescents with the screen time ≥ 2 h a day were more likely to be overweight/obese compared to those with screen time < 2 h a day (OR = 1.67). Evidence also demonstrated that obese children are less physically active compared to non-obese [50,51]. A negative cross-sectional relationship exists between MVPA and childhood obesity [52]. Therefore, adequate MVPA level is suggested as a fundamental goal of pediatric obesity prevention and treatment [53].

Several studies suggested that high birth weight and caesarean section were related to elevated risk of childhood obesity [54,55,56]. Results of the present study support the previous findings. High birth weight and caesarean section were associated with higher risk of excess weight, excess adiposity and AO. Results from a 12-country study demonstrated that ORs of childhood excess weight were significantly higher among children whose birth weights were 3500–3999 g (OR = 1.45), and over 4000 g (OR = 2.08), compared to those with birth weight from 2500 to 2999 g. The positive relationship between birth weight and risk of obesity was observed in girls, whereas a U-shaped association in boys [57]. Meta-analysis indicated a moderately strong association between caesarean section and later obesity [58]. However, other studies reported that caesarean section might elevate the risk of obesity at age 2 years but not at age 6–10 years [59]. In contrast Vehapoglu et al. reported that caesarean section delivery was not associated with increased risk of obesity in children aged 2–14 years [60].

The response rate in present study was about 90.1 %, however there is lack of basic information (sex, age, etc.) on children of which parents have not agreed to participate in project. Therefore it was difficult to examine possible bias of sampling what should be considered as the limitation of this study.

Our findings could be used to develop future public health actions to identify vulnerable subgroups and to prioritize initiatives aiming to prevent and treatment of childhood obesity.

Although our models included many factors contributing to childhood obesity, objective data regarding the child’s activity level and parental obesity were not available. Moreover, data on birth weight were retrospectively obtained from their parents using a questionnaire.

## 5. Conclusions

Parental obesity, screen time, MVPA, and birth weight were significant determinant of excess weight, excess adiposity, and AO in Polish preschool children. Changing modifiable factors associated with obesity in preschool children could reduce the child’s risk for obesity.

## Figures and Tables

**Table 1:** Prevalence of obesity defined based BMI, BFP, and WC percentiles.

Body Mass Classification	Total Sample (*n* = 1172)	Boys (*n* = 600)	Girls (*n* = 572)	*p*
**According to BMI percentiles**				
Underweight	113 (9.6)	41 (6.8)	72 (12.6)	**0.005**
Normal	930 (79.4)	497 (82.8)	433 (75.7)	
Overweight	78 (6.7)	39 (6.5)	39 (6.8)	
Obesity	51 (4.4)	23 (3.8)	28 (4.9)	
**According to BFP percentiles**				
Underfat	38 (3.2)	4 (0.7)	34 (5.9)	**<0.001**
Normal	638 (54.4)	307 (51.2)	331 (57.9)	
Overfat	263 (22.4)	158 (26.3)	105 (18.4)	
Obesity	233 (19.9)	131 (21.8)	102 (17.8)	
**According to WC percentiles**				
Normal	1023 (87.3)	536 (89.3)	487 (85.1)	**0.035**
AO	149 (12.7)	64 (10.7)	85 (14.9)	

Data are expressed as n (%), AO—abdominal obesity, BFP—body fat percentage, BMI—body mass index, WC—waist circumference; significant associations are highlighted in bold.

**Table 2:** Distribution of potential childhood obesity risk factors according to sex.

Variables	Sex			Total Sample (*n* = 1172)
	Boys (n = 600)	Girls (n = 572)	*p*	
**Place of residence**				
Urban *	584 (97.3)	539 (94.2)	**0.008**	1123 (95.8)
Rural *	16 (2.7)	33 (5.8)		49 (4.2)
**Mother’s education**				
High school/University *	466 (77.7)	464 (81.1)	0.149	930 (79.4)
Middle school *	134 (22.3)	108 (18.9)		242 (20.6)
**Father’s education**				
High school/University *	330 (55.0)	354 (61.9)	**0.018**	684 (58.4)
Middle school *	270 (45.0)	218 (38.1)		488 (41.6)
**Socio-economic status**				
High *	566 (94.3)	541 (94.6)	0.899	1107 (94.5)
Middle or low *	34 (5.7%)	31 (5.4)		65(5.5)
**Parental obesity**				
None *	501 (83.5)	484 (84.6)	0.342	985 (84.1)
Father *	38 (6.3)	45 (7.9)		83 (7.1)
Mother *	42 (7.0)	30 (5.2)		72 (6.1)
Both parents *	19 (3.2)	13 (2.3)		32 (2.7)
**Screen time**				
<60 min daily *	341 (56.8)	352 (61.5)	0.256	693 (59.1)
60–120 min daily *	222 (37.0)	190 (33.2)		412 (35.2)
>120 min daily *	37 (6.2)	30 (5.3)		67 (5.7)
**60 min of MVPA**				
Everyday *	175 (29.2)	131 (22.9)	**0.032**	306 (26.1)
2–3 times a week *	287 (47.8)	289 (50.5)		576 (49.1)
Once a week *	86 (14.3)	108 (18.9)		194 (16.6)
Less than once a week *	52 (8.7)	44 (7.7)		96 (8.2)
**Birth weight** (g) ^†^	3400.7 ± 526.5	3261.2 ± 555.8	**<0.001**	3332.6 ± 545.3
<2500 *	20 (3.4)	46 (8.0)	**0.001**	66 (5.6)
2500–4000 *	524 (87.3)	486 (85.0)		1010 (86.2)
>4000 *	56 (9.3)	40 (7.0)		96 (8.2)
**Birth**				
In term **	502 (83.7)	477 (83.4)	0.937	979 (83.5)
Preterm *	98 (16.3)	95 (16.6)		193 (16.5)
**Delivery**				
Natural *	275 (45.8)	271 (47.4)	0.599	546 (46.6)
Caesarean section *	325 (54.2)	301 (52.6)		626 (53.4)

Significant associations are highlighted in bold; data are expressed as: * n (%), ^†^ mean ± SD, MVPA—moderate-to vigorous physical activity.

**Table 3:** Factors associated with excess weight-logistic regression model.

Variables	BMI Classification		Unadjusted OR (95% CI)	*p*	Adjusted OR ^a^ (95% CI)	*p*
	Normal Weight * (*n* = 1043)	Excess Weight * (*n* = 129)				
**Place of residence**						
Urban	997 (95.6)	126 (97.7)	REF	REF	REF	REF
Rural	46 (4.4)	3 (2.3)	0.52 (0.16–1.68)	0.273	0.50 (0.15–1.64)	0.253
**Mother’s education**						
High school/University	836 (80.2)	94 (72.9)	REF	REF	REF	REF
Middle school	207 (19.8)	35 (27.1)	1.50 (0.99–2.28)	0.055	1.52 (1.00–2.30)	0.051
**Father’s education**						
High school/University	626 (60.0)	58 (45.0)	REF	REF	REF	REF
Middle school	417 (40.0)	71 (55.0)	1.84 (1.27–2.66)	**<0.001**	1.86 (1.29–2.70)	**<0.001**
**Socio-economic status**						
High	986 (94.5)	121 (93.8)	REF	REF	REF	REF
Middle or low	57 (5.5)	8 (6.2)	1.14 (0.53–2.45)	0.730	1.14 (0.53–2.46)	0.730
**Parental obesity**						
None	895 (85.8)	90 (69.8)	REF	REF	REF	REF
Father	70 (6.7)	13 (10.1)	1.85 (0.98–3.47)	0.056	1.83 (0.97–3.44)	0.060
Mother	57 (5.5)	15 (11.6)	2.62 (1.42–4.81)	**0.002**	2.66 (1.44–4.89)	**0.002**
Both parents	21 (2.0)	11 (8.5)	5.21 (2.43–11.15)	**<0.001**	5.31 (2.48–11.40)	**<0.001**
**Screen time**						
<60 min daily	624 (59.8)	69 (53.5)	REF	REF	REF	REF
60–120 min daily	364 (34.9)	48 (37.2)	1.19 (0.81–1.76)	0.377	1.20 (0.81–1.78)	0.357
>120 min daily	55 (5.3)	12 (9.3)	1.98 (1.00–3.86)	**0.048**	2.00 (1.01–3.92)	**0.045**
**60 min of MVPA**						
Everyday	280 (26.8)	26 (20.2)	REF	REF	REF	REF
2–3 times per week	515 (49.4)	61 (47.3)	1.28 (0.79–2.06)	0.322	1.26 (0.78–2.05)	0.342
1 time per week	171 (16.4)	23 (17.8)	1.47 (0.81–2.65)	0.206	1.44 (0.79–2.61)	0.228
Less than once a week	77 (7.4)	19 (14.7)	2.66 (1.40–5.05)	**0.003**	2.65 (1.39–5.04)	**0.003**
**Birth weight (g)**						
<2500	59 (5.7)	7 (5.4)	1.13 (0.50–2.54)	0.768	1.08 (0.48–2.45)	0.845
2500–4000	914 (87.6)	96 (74.4)	REF	REF	REF	REF
>4000	70 (6.7)	26 (20.2)	3.54 (2.15–5.81)	**<0.001**	3.59 (2.18–5.90)	**<0.001**
**Birth**						
In term	867 (83.1)	112 (86.8)	REF	REF	REF	REF
Preterm	176 (16.9)	17 (13.2)	0.75 (0.44–1.28)	0.287	0.75 (0.43–1.27)	0.286
**Delivery**						
Natural	499 (47.8)	47 (36.4)	REF	REF	REF	REF
Caesarean section	544 (52.2)	82 (63.6)	1.60 (1.10–2.34)	**0.015**	1.61 (1.10–2.35)	**0.014**

Significant associations are highlighted in bold; * data are expressed as: n (%); OR (95% CI)—odds ratio with a 95% confidence interval; REF—reference category; ^a^—the model adjusted for gender; BMI—body mass index; MVPA—moderate-to vigorous physical activity.

**Table 4:** Factors associated with excess adiposity-logistic regression model.

Variables	BFP Classification		Unadjusted OR (95% CI)	*p*	Adjusted OR ^a^ (95% CI)	*p*
	No Excess Adiposity * (*n* = 676)	Excess Adiposity * (*n* = 496)				
**Place of residence**						
Urban	643 (95.1)	480 (96.8)	REF	REF	REF	REF
Rural	33 (4.9)	16 (3.2)	0.65 (0.35–1.19)	0.165	0.67 (0.35–1.26)	0.217
**Mother’s education**						
High school/University	539 (79.7)	391 (78.8)	REF	REF	REF	REF
Middle school	137 (20.3)	105 (21.2)	1.06 (0.79–1.41)	0.706	1.10 (0.81–1.48)	0.538
**Father’s education**						
High school/University	410 (60.7)	274 (55.2)	REF	REF	REF	REF
Middle school	266 (39.3)	222 (44.8)	1.25 (0.99–1.58)	0.064	1.30 (1.02–1.67)	**0.034**
**Socio-economic status**						
High	639 (94.5)	468 (94.4)	REF	REF	REF	REF
Middle or low	37 (5.5)	28 (5.6)	1.03 (0.62–1.71)	0.899	1.21 (0.71–2.05)	0.480
**Parental obesity**						
None	597 (88.3)	388 (78.2)	REF	REF	REF	REF
Father	39 (5.8)	44 (8.9)	1.74 (1.11–2.72)	**0.016**	1.85 (1.16–2.95)	**0.010**
Mother	32 (4.7)	40 (8.1)	1.92 (1.19–3.11)	**0.008**	1.95 (1.18–3.23)	**0.009**
Both parents	8 (1.2)	24 (4.8)	4.62 (2.05–10.11)	**<0.001**	4.65 (2.01–10.71)	**<0.001**
**Screen time**						
<60 min daily	425 (62.9)	268 (54.0)	REF	REF	REF	REF
60–120 min daily	218 (32.2)	194 (39.1)	1.41 (1.10–1.81)	**0.006**	1.47 (1.14–1.91)	**0.003**
>120 min daily	33 (4.9)	34 (6.9)	1.63 (0.99–2.70)	0.056	1.93 (1.14–3.28)	**0.014**
**60 min of MVPA**						
Everyday	192 (28.4)	114 (23.0)	REF	REF	REF	REF
2–3 times per week	330 (48.8)	246 (49.6)	1.26 (0.94–1.67)	0.117	1.36 (1.01–1.83)	**0.045**
1 time per week	112 (16.6)	82 (16.5)	1.26 (0.87–1.81)	0.226	1.32 (0.90–1.94)	0.160
Less than once a week	42 (6.2)	54 (10.9)	2.16 (1.36–3.45)	**<0.001**	2.51 (1.54–4.08)	**<0.001**
**Birth weight**						
<2500	40 (5.9)	26 (5.2)	0.93 (0.56–1.55)	0.786	0.96 (0.57–1.64)	0.895
2500–4000	595 (88.0)	415 (83.7)	REF	REF	REF	REF
>4000	41 (6.1)	55 (11.1)	1.92 (1.26–2.94)	**0.002**	2.08 (1.34–3.24)	**<0.001**
**Birth**						
In term	561 (83.0)	418 (84.3)	REF	REF	REF	REF
Preterm	115 (17.0)	78 (15.7)	0.91 (0.66–1.24)	0.558	0.89 (0.64–1.24)	0.494
**Delivery**						
Natural	336 (49.7)	210 (42.3)	REF	REF	REF	REF
Caesarean section	340 (50.3)	286 (57.7)	1.35 (1.07–1.70)	**0.013**	1.29 (1.02–1.65)	**0.037**

Significant associations are highlighted in bold; * data are expressed as: n (%); OR (95% CI)—odds ratio with a 95% confidence interval; REF—reference category; ^a^—the model adjusted for gender; BFP—body fat percentage; MVPA—moderate-to vigorous physical activity.

**Table 5:** Factors associated with AO-logistic regression model.

Variables	WC Classification		Unadjusted OR (95% CI)	*p*	Adjusted OR ^a^ (95% CI)	*p*
	Normal * (*n* = 1023)	AO * (*n* = 149)				
**Place of residence**						
Urban	982 (96.0)	141 (94.6)	REF	REF	REF	REF
Rural	41 (4.0)	8 (5.4)	1.36 (0.62–2.96)	0.440	1.26 (0.57–2.75)	0.569
**Mother’s education**						
High school/University	827 (80.8)	103 (69.1)	REF	REF	REF	REF
Middle school	196 (19.2)	46 (30.9)	1.88 (1.29–2.76)	**<0.001**	1.97 (1.34–2.90)	**<0.001**
**Father’s education**						
High school/University	606 (59.2)	78 (52.3)	REF	REF	REF	REF
Middle school	417 (40.8)	71 (47.7)	1.32 (0.94–1.87)	0.112	1.39 (0.98–1.96)	0.065
**Socio-economic status**						
High	972 (95.0)	135 (90.6)	REF	REF	REF	REF
Middle or low	51 (5.0)	14 (9.4)	1.98 (1.06–3.67)	**0.031**	2.09 (1.12–3.91)	**0.020**
**Parental obesity**						
None	877 (85.7)	108 (72.5)	REF	REF	REF	REF
Father	66 (6.5)	17 (11.4)	2.09 (1.18–3.70)	**0.011**	2.05 (1.16–3.63)	**0.014**
Mother	59 (5.8)	13 (8.7)	1.79 (0.95–3.37)	0.072	1.85 (0.62–2.96)	0.059
Both parents	21 (2.1)	11 (7.4)	4.25 (2.00–9.06)	**<0.001**	4.39 (2.05–9.41)	**<0.001**
**Screen time–week day**						
<60 min daily	613 (59.9)	80 (53.7)	REF	REF	REF	REF
60–120 min daily	356 (34.8)	56 (37.6)	1.20 (0.84–1.74)	0.317	1.24 (0.86–1.80)	0.244
>120 min daily	54 (5.3)	13 (8.7)	1.84 (0.96–3.53)	0.064	1.99 (1.03–3.82)	**0.040**
**60 min of MVPA**						
Everyday	271 (26.5)	35 (23.5)	REF	REF	REF	REF
2–3 times per week	510 (49.9)	66 (44.2)	1.00 (0.65–1.55)	0.440	0.97 (0.63–1.51)	0.904
1 time per week	165 (16.1)	29 (19.5)	1.38 (0.81–2.34)	0.440	1.30 (0.76–2.21)	0.340
Less than once a week	77 (7.5)	19 (12.8)	1.91 (1.03–3.53)	**0.039**	1.92 (1.04–3.56)	**0.038**
**Birth weight**						
< 2500	55 (5.4)	11 (7.4)	1.64 (0.83–3.22)	0.154	1.47 (0.74–2.92)	0.268
2500–4000	900 (88.0)	110 (73.8)	REF	REF	REF	REF
> 4000	68 (6.6)	28 (18.8)	3.37 (2.08–5.46)	**<0.001**	3.56 (2.19–5.80)	**<0.001**
**Birth**						
In term	854 (83.5)	125 (83.9)	REF	REF	REF	REF
Preterm	169 (16.5)	24 (16.1)	0.97 (0.61–1.55)	0.899	0.96 (0.60–1.54)	0.878
**Delivery**						
Natural	487 (47.6)	59 (39.6)	REF	REF	REF	REF
Caesarean section	536 (52.4)	90 (60.4)	1.39 (0.98–1.97)	0.068	1.38 (0.97–1.96)	0.074

Significant associations are highlighted in bold; * data are expressed as: n (%); OR (95% CI)—odds ratio with a 95% confidence interval; REF—reference category; ^a^—the model adjusted for gender; AO—abdominal obesity; MVPA—moderate-to vigorous physical activity.

## Data Availability

The data presented in this study are available on request from the corresponding author.

## Supplementary Materials

The following are available online at https://www.mdpi.com/2077-0383/10/4/790/s1, Figure S1: The prevalence and risk factors of excess body weight among Polish preschoolers, Figure S2: The prevalence and risk factors of excess body adiposity among Polish preschoolers, Figure S3: The prevalence and risk factors of abdominal adiposity among Polish preschoolers.
