# Supplementary material for: Prevalence of Overweight, Obesity, Abdominal Obesity, and Obesity-Related Risk Factors in Polish Preschool Children: A Cross-Sectional Study
Source: J Clin Med. 2021 Feb 16;10(4):790. doi: 10.3390/jcm10040790 (PMC7920301; doi:10.3390/jcm10040790)
Supplement: Supplementary file 1 [file jcm-10-00790-s001.pdf]

Journal of
Clinical Medicine

MDPI

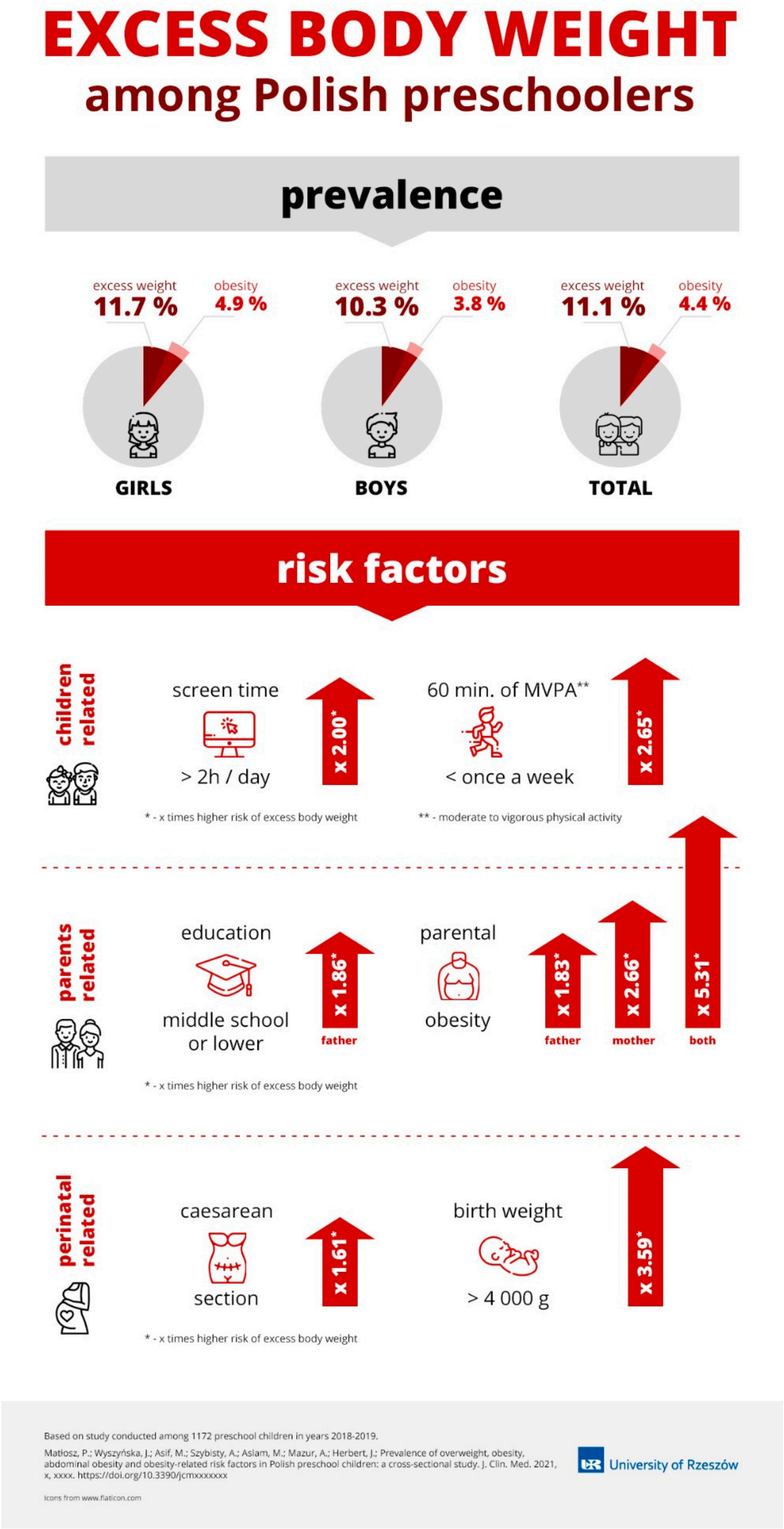

Figure S1. The prevalence and risk factors of excess body weight among Polish preschoolers.

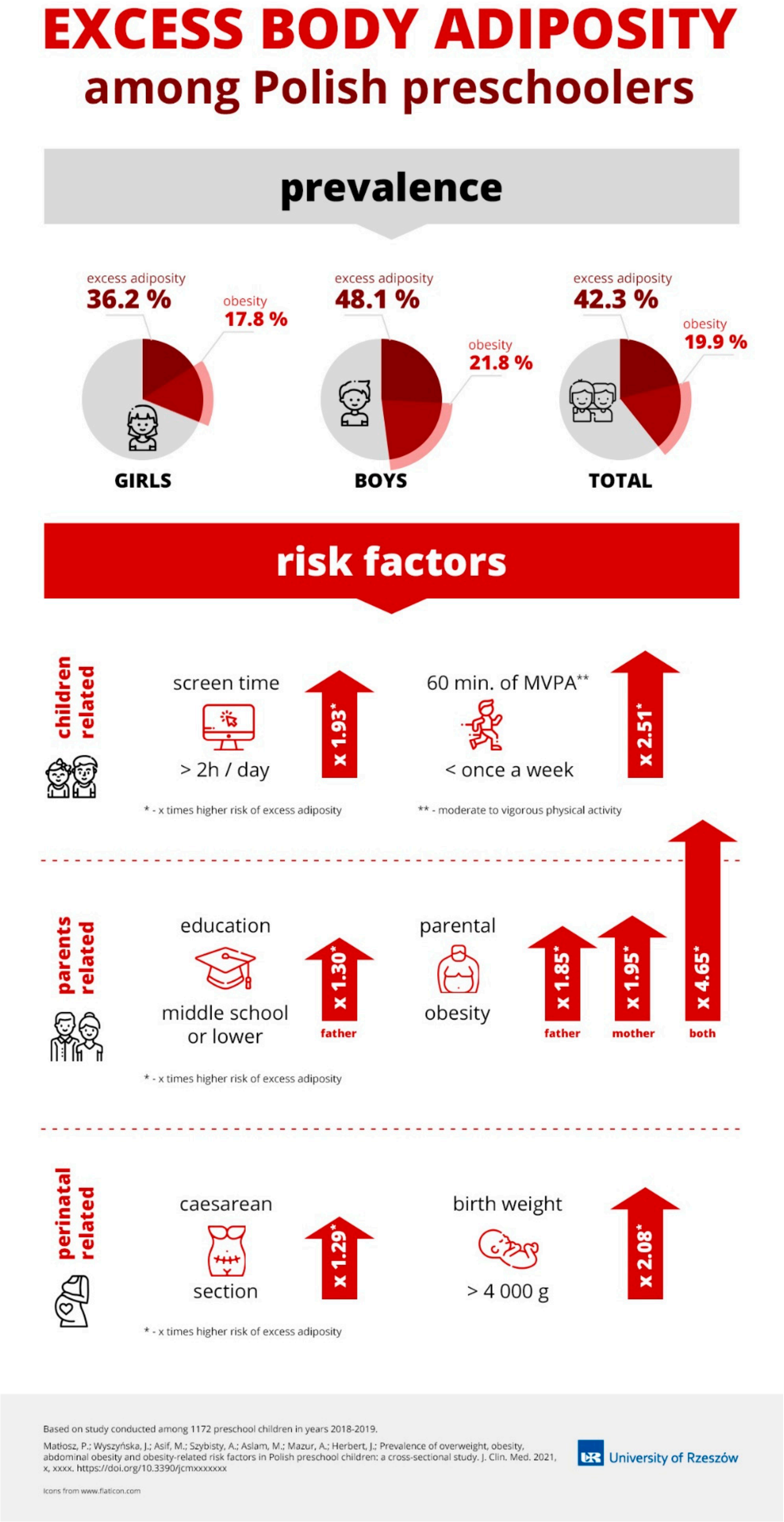

Figure S2. The prevalence and risk factors of excess body adiposity among Polish preschoolers.

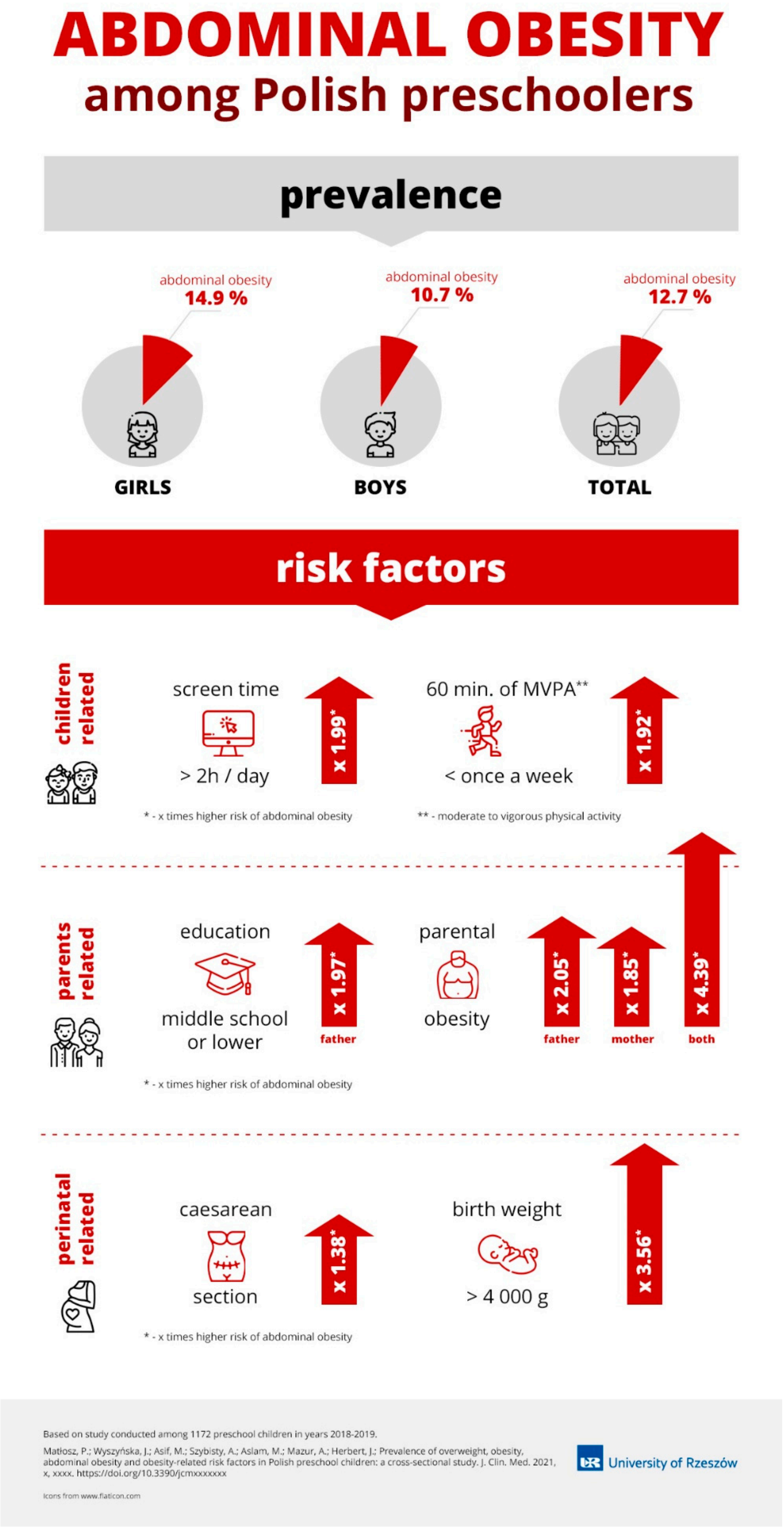

Figure S3. The prevalence and risk factors of abdominal adiposity among Polish preschoolers.
